# Epigenetic Dysregulation of Trophoblastic Gene Expression in Gestational Trophoblastic Disease

**DOI:** 10.3390/biomedicines9121935

**Published:** 2021-12-17

**Authors:** Zoltan Szabolcsi, Amanda Demeter, Peter Kiraly, Andrea Balogh, Melissa L. Wilson, Jennifer R. King, Szabolcs Hetey, Zsolt Gelencser, Koji Matsuo, Beata Hargitai, Paulette Mhawech-Fauceglia, Petronella Hupuczi, Andras Szilagyi, Zoltan Papp, Lynda D. Roman, Victoria K. Cortessis, Nandor Gabor Than

**Affiliations:** 1 Systems Biology of Reproduction Research Group, Research Centre for Natural Sciences, Institute of Enzymology, H-1117 Budapest, Hungary; szabzoltan@gmail.com (Z.S.); ammiwa@gmail.com (A.D.); kiraly.2.peter@ttk.hu (P.K.); balogh.andrea@ttk.hu (A.B.); heteysz@gmail.com (S.H.); gelzsolt@gmail.com (Z.G.); szilagyi.andras@ttk.hu (A.S.); 2 Department of Population and Public Health Sciences, University of Southern California, Los Angeles, CA 90033, USA; melisslw@usc.edu (M.L.W.); victoria.cortessis@med.usc.edu (V.K.C.); 3 Department of Obstetrics and Gynecology, Keck School of Medicine, University of Southern California, Los Angeles, CA 90033, USA; jennyrenaeking@gmail.com (J.R.K.); koji.matsuo@med.usc.edu (K.M.); lroman@usc.edu (L.D.R.); 4 Birmingham Women’s NHS Foundation Trust, University of Birmingham, Birmingham B15 2TG, UK; beatahargitai@hotmail.com; 5 Department of Pathology, Keck School of Medicine, University of Southern California, Los Angeles, CA 90033, USA; pfauceglia@hotmail.com; 6 Maternity Private Clinic, H-1126 Budapest, Hungary; hupuczi.petronella@maternity.hu (P.H.); pzorvosihetilap@maternity.hu (Z.P.); 7 Department of Obstetrics and Gynecology, Semmelweis University, H-1088 Budapest, Hungary; 8 First Department of Pathology and Experimental Cancer Research, Semmelweis University, H-1085 Budapest, Hungary

**Keywords:** choriocarcinoma, hydatidiform mole, gestational trophoblastic disease, placental-specific gene, systems biology, trophoblast differentiation

## Abstract

Gestational trophoblastic diseases (GTDs) have not been investigated for their epigenetic marks and consequent transcriptomic changes. Here, we analyzed genome-wide DNA methylation and transcriptome data to reveal the epigenetic basis of disease pathways that may lead to benign or malignant GTDs. RNA-Seq, mRNA microarray, and Human Methylation 450 BeadChip data from complete moles and choriocarcinoma cells were bioinformatically analyzed. Paraffin-embedded tissues from complete moles and control placentas were used for tissue microarray construction, DNMT3B immunostaining and immunoscoring. We found that DNA methylation increases with disease severity in GTDs. Differentially expressed genes are mainly upregulated in moles while predominantly downregulated in choriocarcinoma. DNA methylation principally influences the gene expression of villous trophoblast differentiation-related or predominantly placenta-expressed genes in moles and choriocarcinoma cells. Affected genes in these subsets shared focal adhesion and actin cytoskeleton pathways in moles and choriocarcinoma. In moles, cell cycle and differentiation regulatory pathways, essential for trophoblast/placental development, were enriched. In choriocarcinoma cells, hormone biosynthetic, extracellular matrix-related, hypoxic gene regulatory, and differentiation-related signaling pathways were enriched. In moles, we found slight upregulation of DNMT3B protein, a developmentally important de novo DNA methylase, which is strongly overexpressed in choriocarcinoma cells that may partly be responsible for the large DNA methylation differences. Our findings provide new insights into the shared and disparate molecular pathways of disease in GTDs and may help in designing new diagnostic and therapeutic tools.

## 1. Introduction

Gestational trophoblastic disease (GTD) refers to a spectrum of disorders that can arise in healthy, young women if dispermic conception occurs, resulting in molar pregnancy, or if trophoblastic tissue remains in the uterus following apparently normal pregnancy [1,2]. GTD is readily treated if detected early, when complete evacuation achieves cure and preserves fertility [3]. However, delayed detection can increase the risk of complications of varying severity, which include uterine muscle invasion, bleeding that may necessitate hysterectomy and concomitant loss of fertility, and metastatic disease that can require multi-agent chemotherapy and which in turn confers elevated risk of severe morbidity and death [3]. Early detection of GTD is therefore paramount, yet delays occur in both medically underserved women and in those who receive adequate prenatal care.

The spectrum of GTD ranges from relatively common products of abnormal conception with recognized malignant potential to rarer forms of frank malignancy that if allowed to progress can have high case fatality [4]. Hydatidiform moles, the form of GTD that arise earliest in gestation, have well characterized origins in dispermic conception and reportedly complicate 1–8 pregnancies per 1000 [5,6,7]. In women of European descent, this proportion was until recent years 1–2 per 1000 pregnancies, with the more benign incomplete moles predominating [8]. However, because the more aggressive complete moles are more common in pregnancies of women younger than 20 or older than 40, and the demographic shift toward older ages at childbearing has caused the overall proportion of pregnancies complicated by moles to increase, complete moles are now most common [9].

Incomplete moles are distinguished from complete moles by karyotypic analysis revealing triploid state, and by genotypic analysis identifying genetic material of both maternal and paternal origin [10]. Complete moles, which are diploid and bear only genetic material of paternal origin, can have either heterozygous genotypic state consistent with the fertilization of a postulated “empty ovum” by two sperm, or exclusively homozygous genotype, proposed to arise from the duplication of a single sperm following fertilization of an “empty ovum” [10]. The empty ova proposed in these models may originate by nondisjunction, creating aneuploid maternal pronuclei followed by loss of maternal material [11]. This model could in theory explain elevated risk of complete moles in very young women and those of advanced age, because aneuploid conceptuses are more often reported in women of these ages.

Gestational trophoblastic neoplasia (GTN) can arise either after molar pregnancy or after healthy pregnancy. The risk of GTN is particularly high in pregnancies complicated by complete mole, with a risk of GTN of 15–20% of such cases [8]. Although cure can be achieved in over 90% of GTN, unrecognized and misdiagnosed GTN can result in unnecessarily increased maternal morbidity and mortality [12].

Molar pregnancies arise in conceptuses of disordered epigenetic state, and the abnormal DNA methyl marks recognized in these tissues [13] and similar disruptions were more recently confirmed in advanced choriocarcinoma [14,15,16]. The DNA content of hydatidiform moles shows that the pathology of molar trophoblasts has a fundamentally epigenetic rather than genetic basis. Indeed, pathology is more severe in complete moles, which carry the normal number of 46 human chromosomes, compared to triploid incomplete moles, which have 69 chromosomes. However, complete moles carry only paternal epigenetic marks at differentially methylated regions responsible for genomic imprinting, while incomplete moles carry two paternal and one maternal copies [10,11]. Some recent studies attempted to characterize the methyl marks of genomic DNA in choriocarcinoma cell lines [15,17]. However, choriocarcinoma tissues have not been investigated for their epigenetic marks at the genome level.

Here, we compared the epigenetic state at the DNA methylation level in cells from hydatidiform moles and choriocarcinoma to normal first trimester trophoblasts and placenta. In parallel, we evaluated patterns of genome-wide mRNA expression to investigate the epigenetic basis of transcriptomic differences between these cell types, in an effort to recognize the pathways that may distinguish benign from malignant GTDs.

## 2. Materials and Methods

### 2.1. Tissue Samples

Gestational trophoblastic disease (GTD) tissue samples had been collected during usual care of women who underwent treatment for GTD at the Department of Obstetrics, Keck School of Medicine, University of Southern California (Los Angeles, CA, USA). We included 17 cases of complete mole for which good quality tissue blocks were available, and used immunohistochemistry to confirm loss of p57 expression as reported in our previous study [18] (Table A1). Placing no restriction on stage of gestation, we found the pregnancies to be between 5 and 15 weeks of gestation according to ultrasound scans; patients with multiple pregnancies were excluded.

Control samples of first trimester placental tissue (*n* = 24) matched by gestational age to GTD samples had been collected prospectively at the Maternity Private Clinic (Budapest, Hungary). We selected formalin-fixed, paraffin-embedded samples from pregnancies voluntarily terminated between 5 and 12 weeks of gestation according to ultrasound scans, excluding multiple pregnancies (Table A1).

### 2.2. Histopathologic Evaluation of Tissues

Samples of tissue from both first trimester placenta and GTD had been fixed in 10% neutral-buffered formalin and embedded in paraffin (FFPE). Five-µm sections were cut from tissue blocks, stained with hematoxylin and eosin (H&E), and examined using light microscopy by a perinatal pathologist (B.H.) and a pathologist with expertise in gynecologic malignancies (P.M.-W.) to identify regions of each tissue block to sample for tissue microarrays. Both examiners, blinded to patients’ clinical information except gestational age, histopathologically examined placental and GTD samples using a standard perinatal pathological protocol and previously published diagnostic criteria [19,20,21].

### 2.3. Tissue Microarray (TMA) Construction, Immunohistochemistry, Immunoscoring

As described in [22], TMAs were constructed, each consisting of three cylindrical cores of 2 mm diameter from each sample of first trimester FFPE placental and GTD tissue specimens. Cores from the same sample were transferred into recipient paraffin blocks adjacent to each other using an automated tissue arrayer (TMA Master II, 3DHISTECH Ltd., Budapest, Hungary). Each recipient block also contained liver cores representing negative control and a third trimester placenta core as positive control material.

To interrogate expression at the protein level, we conducted a series of immunostains on p57 and DNMT3B proteins to be determined on TMA slides. Five-µm sections from each TMA were cut and then stained by standard H&E or immunostained as follows. Sections were prepared for immunostaining by being cut from TMAs, placed on silanized slides, deparaffinized using xylene and rehydrated in graded alcohol series. Endogenous peroxidase blocking was performed using 10% H_2_O_2_ for 20 min, and antigen retrieval was performed using Tris-EDTA pH9 buffer for 32 min at 100 °C. Sections were then blocked for 10 min (Novolink) and incubated with specific antibodies as outlined in Table A2. After three washes, the Novolink Polymer Detection System (Leica-Novocastra, Wetzlar, Germany) was used as secondary antibody (30 min, room temperature), followed by three washes, and detected using 3,3-diaminobenzidine in 1:20 dilution. Finally, sections were counterstained with hematoxylin and were mounted after dehydration.

TMA immunostaining was scored semi-quantitatively for p57 as detailed in [18]. TMA immunostaining for DNMT3B was scored automatically by NuclearQuant image analysis module of Pannoramic Viewer (3DHISTECH Ltd., Budapest, Hungary) using basic settings. The cytotrophoblastic and syncytiotrophoblastic layers were selected and 2–6 annotations were performed from 2–3 cores from each donor.

### 2.4. High-Dimensional Molecular Data

We obtained DNA methylation and gene expression data detailed in Supplementary Table S1, from the Gene Expression Omnibus (GEO) database. In brief, we extracted Infinium Human Methylation 450 array raw data for first trimester villous trophoblast from GSE93208 (*n* = 19 samples), first trimester placental data from GSE66210 (*n* = 12 samples), data for complete hydatidiform mole from GSE52576 (*n* = 4 samples), and data for the JAR/JEG-3 choriocarcinoma cell line from GSE68379 (*n* = 2 samples). Gene expression data were extracted for villous trophoblast from GSE9773 (*n* = 5 samples), complete hydatidiform mole from GSE138250 (*n* = 4 samples), placental tissue from GSE138250 (*n* = 1 sample) and GSE109082 (*n* = 39 samples), and for JEG-3 from GSE20510 (*n* = 4 samples) U133A microarrays.

### 2.5. Methylation Data Analysis

We implemented data analyses using packages of the R statistical computing environment and Bioconductor. For DNA methylation analysis we calculated β- and M-values for each methylation site (given as cgIDs), normalizing for the batch effects between studies using the minfi package (v. 1.20.2) [23]. We used the preprocessFunnorm functional normalization algorithm of minfi to adjust between-array technical variations [24].

To compare methylation levels across the four sample groups (villous trophoblast, placenta, complete mole, and choriocarcinoma), we carried out differential methylation (DM) analysis between sample groups by linear modelling using the lmFit and eBayes functions of the limma package (v. 3.30.13) [25]. We used a matrix of M-values as input data for the DM analysis.

We performed hierarchical clustering of all samples based on β-values, using all cgIDs. For clustering, we used the hclust function from the stats package and pvclust function from the pvclust package with Euclidean distances for the distance matrix and ward.D2 method for clustering.

The median of β-values was calculated for each cgID over samples belonging to each tissue type. Finding villous trophoblast to have the lowest level of DNA methylation, we used data from this tissue type as reference level in subsequent analyses. We estimated the difference in DNA methylation between placenta, molar tissues, and choriocarcinoma by subtracting the corresponding median β-values of the cgIDs in these tissues from those in the villous trophoblast. We inferred significance by using false discovery rate (FDR) corrected *p*-values (*q*-values) generated from the DM lists (generated by limma) for each comparison. *q*-values of <0.05 were considered significant.

To examine relationships between differences in DNA methylation and differences in expression, we carried out a separate analysis comparing choriocarcinoma samples with villous trophoblast samples, and mole samples with placenta samples. In this analysis, differentially methylated sites were determined using Student’s or Welch’s *t*-test on M-values, depending on whether variances were found to be equal or unequal based on an F-test for equal variances. Here, *q*-values <0.1 were considered significant.

We annotated the data with the gene symbol for each cgID using the downloadable Illumina data table for Platform GPL13534 from GEO. Further annotations were assigned based on the org.Hs.eg.db package (v. 3.14.0). The start and end positions for each probe and their distances from TSS were identified with the FDb.InfiniumMethylation.hg19 package (v2.2.0).

Differentially methylated sites (cgIDs) were grouped according to the difference (Δβ) in median β-values into 3 categories, mild, moderate, and strong (i.e., 0.125 < |Δβ| ≤ 0.25, 0.25 < |Δβ| ≤ 0.5, and |Δβ| > 0.5) as described in [16,26]. A gene was considered differentially up/downmethylated if any cgID associated with it was found to be differentially up/downmethylated. Genes with ambiguous methylation (i.e., some cgIDs associated with the same gene were upmethylated while others were downmethylated) were excluded from further analyses.

The distribution of hyper- or hypomethylated sites near transcription start sites (TSS) was determined by selecting those cgIDs that are within 1 kbp distance from TSS. Then, we partitioned this 1 kbp interval into 10 segments 100 bp in length and determined for each the frequency of hyper- or hypomethylated sites relative to the total number of cgIDs in the 1 kbp interval.

### 2.6. Analysis of Imprinted Sites

A list of known probes (cgIDs) mapping to imprinted differentially methylated regions was downloaded from humanimprints.net [27]. DNA methylation levels in the villous trophoblast, placenta, complete mole, and choriocarcinoma samples were analyzed separately for maternally and paternally imprinted sites.

### 2.7. mRNA Microarray Analysis

For differential expression (DE) analysis of mRNA levels in villous trophoblasts and JEG-3 choriocarcinoma cells the obtained CEL files were processed in R. Expression intensities were background-corrected, normalized and log_2_ transformed using the default method in the rma function of the affy package [28]. Entrez IDs, gene symbols, and gene names of the probes were determined using the hgu133a.db package. From 22,283 probes on the array, we kept 12,437 unique probes which had Entrez IDs and the lowest adjusted *p*-value among probes for each gene. Differential gene expression was set at FDR-adjusted *p*-value (*q*-value) of <0.1 and absolute fold change of ≥2.

### 2.8. RNAseq Analysis

RNA-Seq data from complete moles and control individuals were obtained from our parallel study [18]. Differential expression was set at a DEseq2 corrected *p*-value (*q* value) of <0.05 and absolute fold change of ≥2.

### 2.9. Comparison of Differential DNA Methylation and Differential mRNA Expression

The DM gene lists were compared to the DE gene lists from two types of gene expression data. To compare choriocarcinoma cells to villous trophoblasts, we used the DE gene list from microarray data (Supplementary Table S2), whereas to compare molar tissues to placentas, we used the DE gene list from RNAseq data [18]. The differential methylation threshold was set at a minimum of 12.5% (i.e., |Δβ| > 0.125) in all instances as described in [16,26]. Gene lists were divided into groups of up- and downmethylated as well as up- and downregulated genes, and various intersections and differences of the resulting sets were taken.

### 2.10. Pathway Analysis

The enrichment of pathways in various sets of genes was determined using the DAVID pathway analysis tool [29]. The functional annotation charts and tables were retrieved for the pathways in the KEGG pathway database [30].

### 2.11. TMA Immunoscoring Analysis

p57 immunoscoring analysis was described in detail in [18]. For DNMT3B immunoscoring analysis, the cytotrophoblastic and syncytiotrophoblastic layers were selected and 2–6 annotations were performed from the 2–3 cores per donor. A score was rendered for each annotation, the mean of the annotations per each core was calculated, and finally the mean score of the cores were calculated for each donor. Group comparisons were then conducted by averaging immunoscores at the level of tissue type. The expression of each protein in complete molar tissue was compared to that in placenta tissue by estimates of mean and standard deviation of immunoscores in each group. We used the Mann–Whitney test, and *p*-values of <0.05 were considered significant.

## 3. Results

**Figure 1:**
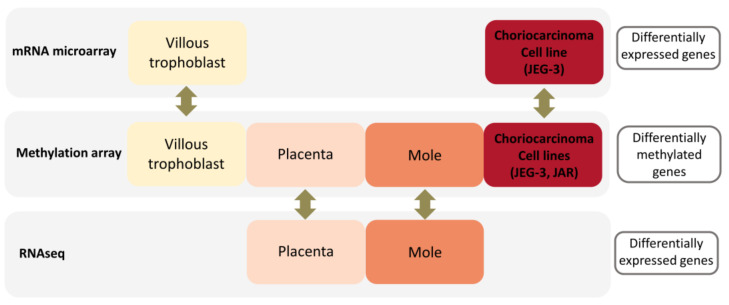
Flowchart of the data analysis. DNA methylation data for four sample groups (villous trophoblast, placenta, complete mole, and choriocarcinoma) were matched with corresponding gene expression data (mRNA microarray for villous trophoblast and choriocarcinoma, and RNAseq for placenta and complete mole).

The analysis had three broad objectives: (1) to identify CpG sites differentially methylated between GTD tissue and normal trophoblast, and between choriocarcinoma and complete hydatidiform moles; (2) to learn whether differential DNA methylation is accompanied by differential expression or mRNA encoded by nearby genes; and (3) to identify processes and pathways disrupted in GTD. A schema of data types compared in the differential methylation and expression analyses are depicted in Figure 1.

### 3.1. Level of DNA Methylation Is Related to Severity of Gestational Trophoblastic Disease

**Figure 2:**
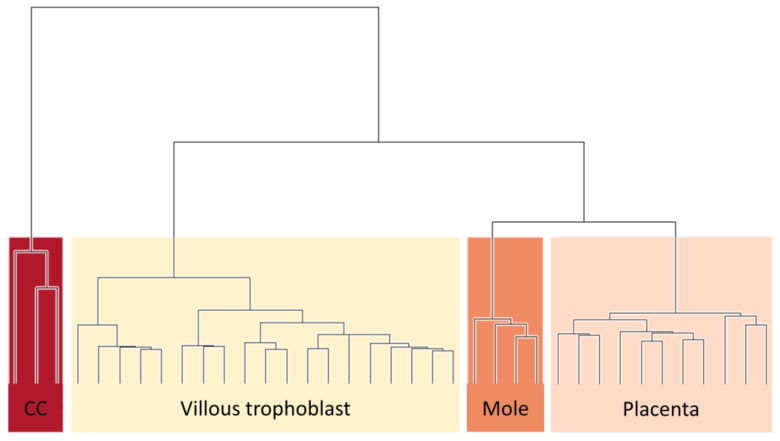
Hierarchical clustering of DNA methylation data perfectly allocates samples of villous trophoblast, placenta, complete mole, and choriocarcinoma cell samples into separate clades. A matrix of Euclidean distances between all samples was calculated from the β values for all cgIDs, and a hierarchical clustering procedure was applied.

Unsupervised cluster analysis of DNA methylation levels clearly separated samples of each type (villous trophoblast, placenta, complete mole, and gestational choriocarcinoma) into distinct clades based on all genes (*n* = 18,740). Moreover, choriocarcinoma cells were clustered most distantly from the villous trophoblast and placental samples, while complete mole samples were sorted more closely to these groups. Complete moles were found to be the sister clade of placental tissues, in accordance with both tissues containing mixed types of cells, in contrast to pure villous trophoblast cells (Figure 2).

Based on the median of β-values of DNA methylation across all cgIDs in all samples of each sample type, villous trophoblast had the lowest degree of methylation (β-median: 0.25), and significantly higher DNA methylation was detected in placenta (0.37), complete moles (0.45), and choriocarcinoma (0.59).

**Figure 3:**
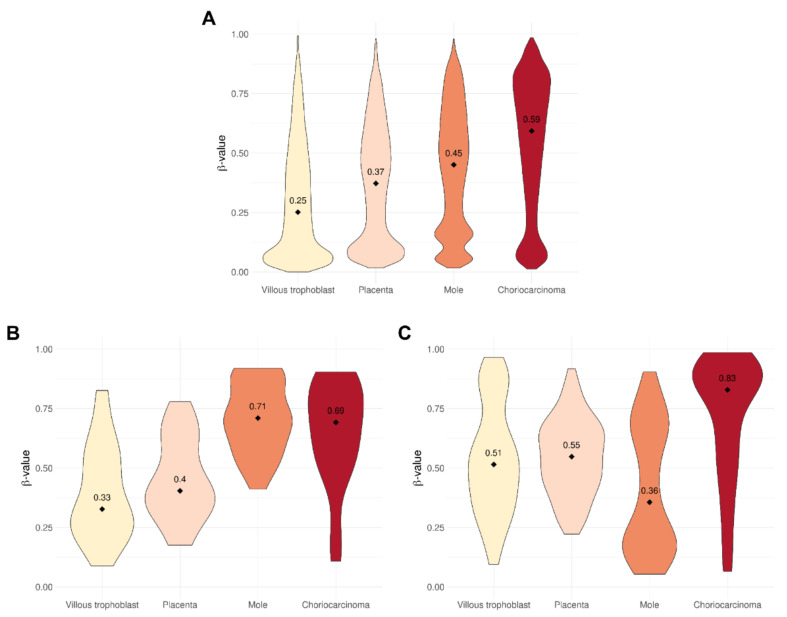
Violin plots show the distribution of cgID β-values for each tissue type. (**A**) Taking into account all cgIDs on the array, DNA methylation was found to be the lowest in villous trophoblast and sequentially higher in placenta, complete mole and choriocarcinoma. (**B**,**C**) The analysis of probes mapping to maternally (**B**, *n* = 139) or paternally (**C**, *n* = 633) imprinted differentially methylated regions showed that, compared to all cgIDs, maternally imprinted cgIDs are more highly methylated both in DNA from moles and choriocarcinoma, while paternally imprinted cgIDs are less methylated in moles but more highly methylated in choriocarcinoma.

Distributions of β-values for unselected loci were visualized as violin (density) plots for each tissue type (Figure 3A). The resulting patterns illustrate greater levels of DNA methylation in the sequence of escalating pathology: normal tissue (villous trophoblast, placenta), complete moles, and choriocarcinoma. Plots for all tissue types demonstrate peaks at very low levels of DNA methylation, while there is a distinct peak at around β = 0.15 in the plot for complete moles, and a broader peak around β = 0.8 in the plot for choriocarcinoma.

Probes mapping to differentially methylated imprint marks had quite different distributions. At the maternally imprinted (typically, paternally expressed) sites (*n* = 139), levels of DNA methylation were higher in both pathologic tissue types than in normal types, and highest by far in complete moles (Figure 3B). By contrast, at paternally imprinted (typically, maternally expressed) sites (*n* = 633), choriocarcinoma cells had far more highly methylated sites than the normal tissue types, while molar tissue demonstrated lower levels than all other types (Figure 3C).

**Figure 4:**
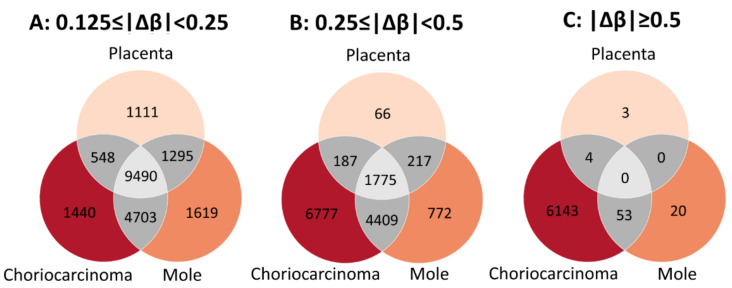
Venn diagrams depict the overlap between differentially methylated genes in gestational trophoblastic disease. The number of genes corresponding to differentially methylated cgIDs at three levels of differential DNA methylation (**A**: 0.125 < |Δβ| ≤ 0.25, **B**: 0.25 < |Δβ| ≤ 0.5, and **C**: |Δβ| > 0.5) are indicated in three sample types (placentas, complete moles and choriocarcinoma).

Next, for each of placenta, molar tissue, and choriocarcinoma, the differentially methylated genes were scored by the level of DNA methylation in relation to level in villous trophoblast samples. Scoring of these genes is reported in Supplementary Table S3. For numerous genes, there were small differences for all three tissue types (Δβ, 0.125–0.25; Figure 4A). Fewer genes demonstrated moderate differences (Δβ, 0.25–0.5), and these were more numerous for pathologic tissue types, particularly choriocarcinoma (Figure 4B). The largest methylation differences (Δβ > 0.5) were almost exclusively found for choriocarcinoma (Figure 4C).

**Figure 5:**
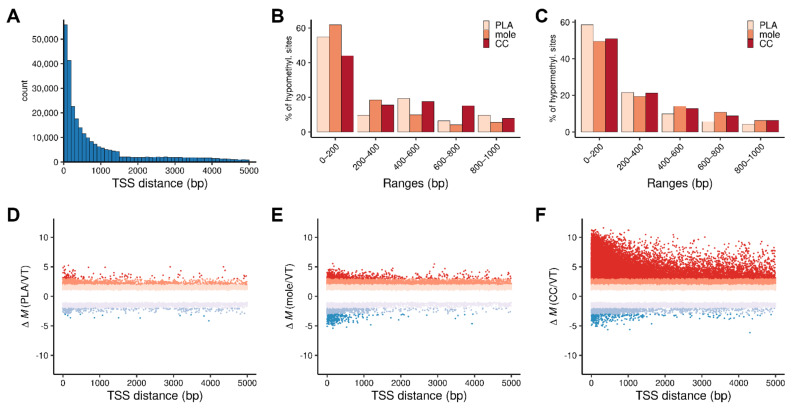
Charts show differential CpG methylation in three tissues compared to villous trophoblast as a function of the distance to the transcription start sites. (**A**) Distribution of cgID positions within 5 kbp from the transcription start sites (TSS). (**B**) Percentage distribution of hypomethylated sites in 200 bp bins within 1 kb from the TSS in the three tissues. (**C**) Same as (**B**) for hypermethylated sites. (**D**–**F**) Scatter plots show methylation levels (ΔM values) of the CpGs in the placenta (**D**), complete moles (**E**), and choriocarcinoma (**F**). Colors indicate the level of hypermethylation (red) and hypomethylation (blue). PLA, placenta; mole, complete mole; CC, choriocarcinoma.

We further characterized differential DNA methylation according to the direction (hypomethylation or hypermethylation of CpGs in placenta, complete mole, or choriocarcinoma compared to villous trophoblast), magnitude (ΔM), and distance of CpG sites from the nearest transcription start site (TSS). CpG islands are mostly found within promoters and first exons, especially in highly expressed genes [31,32]. Accordingly, CpG sites located close to a TSS, particularly within 200 bp, are highly represented on the array (Figure 5A). In all three tissues, differential methylation was most frequently observed at sites within 200 bp from a TSS, where hypomethylation was observed most frequently in complete moles, and hypermethylation most frequently in the placenta (Figure 5B,C). At sites within the remaining 5 kbp from a TSS, differential methylation was more common in the pathologic tissues than in placenta, and hypermethylation was overwhelmingly more frequent and of greater magnitude in choriocarcinoma (Figure 5D–F).

### 3.2. Differentially Expressed Genes Are Mainly Upregulated in Complete Moles While Predominantly Downregulated in Choriocarcinoma

**Figure 6:**
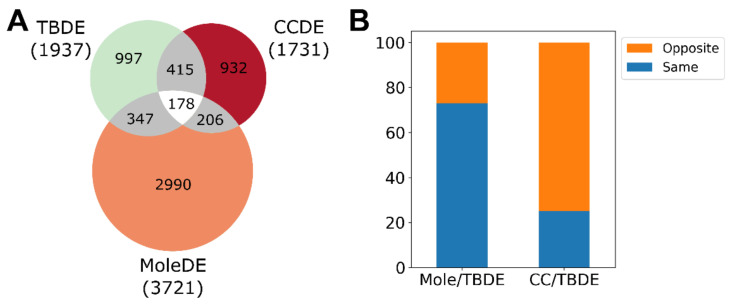
Genes differentially expressed in moles and choriocarcinoma compared to genes differentially expressed during trophoblast differentiation. (**A**) Venn diagram of the genes differentially expressed (DE) in moles, choriocarcinoma (CC) and during trophoblast differentiation (TBDE). (**B**) The fraction of DE genes in moles and in choriocarcinoma with differential expression in the same/opposite direction as during trophoblast differentiation.

We previously compared mRNA expression in complete moles and normal first trimester placenta and reported that among protein coding genes expressed in all samples (14,022), 27% (3729) were differentially expressed. The great majority of these, 72%, were more highly expressed in molar tissue than in placenta. We found the full set of differentially expressed genes to be enriched (*n* = 63, OR = 1.9) with loci that demonstrate placenta-specific expression [26,33]. Most of these (79%, 50/73) were downregulated in molar tissue. [18]. Genes that are differentially regulated during villous trophoblast differentiation were minimally enriched (*n* = 525, OR = 1.15) (Figure 6A). We now report that the expression of 73% of these (*n* = 383) genes differs in molar tissue versus placenta in the same direction as expression characteristically changes during trophoblast differentiation (Figure 6B).

By comparing gene expression reported in JEG-3 choriocarcinoma cells and first trimester trophoblast cells [34,35], we identified 1731 differentially expressed genes. Of these, 1198 (69%) were downregulated in choriocarcinoma. This set of differentially expressed genes is highly enriched (*n* = 107, OR = 15.9) for the loci that demonstrate placenta-specific expression [26,33], and nearly all of these loci (99%, 106/107) were downregulated in choriocarcinoma [18]. In contrast to genes differentially expressed in molar tissue, the set differentially expressed in choriocarcinoma is highly enriched (*n* = 593, OR = 5.69) for genes that are differentially regulated during villous trophoblast differentiation (Figure 6A). Differences in expression of 75% of these genes (*n* = 444) differ in a direction that is opposite to characteristic changes in expression during trophoblast differentiation (Figure 6B), in further contrast to molar tissue. These results indicate that in choriocarcinoma gene regulatory changes strongly interfere with the villous trophoblast differentiation program and negatively affect placental functions to a considerable degree.

**Figure 7:**
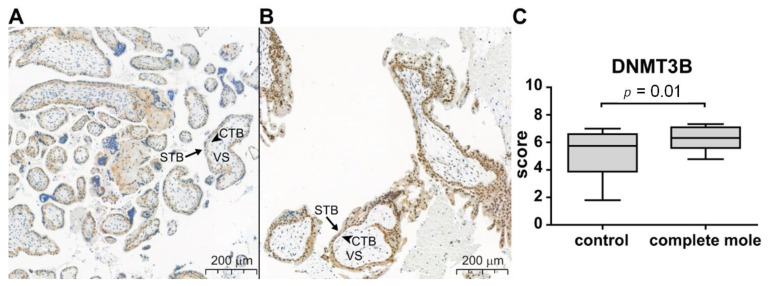
Differential expression of DNMT3B in the villous trophoblast in complete moles and first trimester control placentas. Five-µm-thick first trimester placental sections from normal pregnancy (**A**, control) and complete moles (**B**) were stained for DNMT3B. In control placentas, DNMT3B immunostaining was detectable in the cytoplasm and nuclei of the proliferating villous cytotrophoblasts (CTB, arrowheads) but not in other cells types or the villous stroma (VS). In complete moles, stronger immunostaining was observed in the CTB layer and a weak staining also in the syncytiotrophoblast (STB) (arrows). (**C**) Composite DNMT3B immunoscores (mean ± SEM) for the villous trophoblastic layers in control placentas (*n* = 24) and complete moles (*n* = 17). The unpaired *t*-test was used to compare mean immunoscores between the groups. Representative images, hematoxylin counterstain, 100× magnifications.

In choriocarcinoma cells, an important finding was the significant overexpression (3.12 log_2_ fold-change, *q* = 2.4 × 10^−5^) of DNMT3B, which has the enzyme product that is involved in de novo DNA methylation during development. In complete moles, this gene had only a 10% (0.14 log_2_ fold-change, *q* = 0.5) upregulation. We investigated the DNMT3B protein product in our collection of GTD samples embedded on TMA slides. We chose only those 17 cases who had good quality tissue samples from complete molar pregnancies confirmed by both of our histopathological examinations and negative immunostaining for cyclin-dependent kinase inhibitor p57 (p57). We could not investigate choriocarcinoma tissues due to the lack of good quality tissue. We observed faint villous cytotrophoblastic expression of DNMT3B in control placentas, while stronger cytotrophoblastic staining was detected besides clear syncytiotrophoblastic immunopositivity in complete moles. In accord, the mean composite DNMT3B immunoscores of the villous trophoblast layers were 23% higher in complete moles than in gestational age-matched controls (6.33 ± 0.209 and 5.14 ± 0.347, respectively, *p* = 0.01) (Figure 7A–C).

### 3.3. DNA Methylation Influences Gene Expression of Villous Trophoblast Differentiation-Related or Predominantly Placenta-Expressed Genes

Next, we examined an alleged effect on the differential gene expression mediated by DNA methylation in GTDs. To accomplish this, we matched available DNA methylation and gene expression data for complete moles, choriocarcinoma, first trimester placenta, and villous trophoblasts. All genes were connected to their mRNA expression (fold change values) and differential methylation data (Δβ).

In complete moles, among 1590 differentially expressed genes, 1073 (67%) were found to be hypermethylated (Δβ ≥ 0.125). From these, 341 (32%) were downregulated and 732 (68%) upregulated. Conversely, 517 genes (33%) were found to be hypomethylated (Δβ ≤ −0.125). From these, 406 (78.5%) were found to be upregulated and 111 (21.5%) downregulated (Table 1 and Supplementary Table S4).

In choriocarcinomas, among 1098 differentially expressed genes, 1055 (96%) were found to be hypermethylated (Δβ ≥0.125). From these, 748 (71%) were downregulated and 307 (29%) upregulated. Conversely, 43 genes (4%) were found to be hypomethylated (Δβ ≤ −0.125). From these, 18 (42%) were found to be upregulated and 25 (58%) downregulated (Table 1 and Supplementary Table S4).

**Figure 8:**
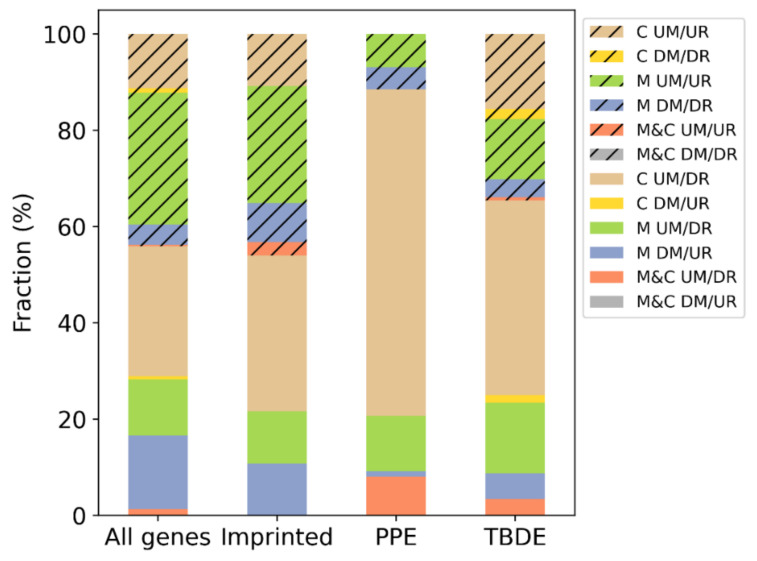
The distribution of differentially DNA methylated and differentially expressed genes in various genes subsets. Subsets of genes were defined based on the direction of DNA methylation and gene expression changes (UM: upmethylated, DM: downmethylated, UR: upregulated, DR: downregulated) in moles (M), choriocarcinoma (C), and their intersections (M&C). The distribution is shown for all genes, imprinted genes, predominantly placenta expressed (PPE) genes, and villous trophoblast differentiation related (TBDE) genes. Striped subsets belong to the genes regulated contrarily to our expectation based on the methylation change (UM/UR, DM/DR).

These results suggest that in complete moles, the gene expression in general is not affected by the DNA methylation status at the relatively low threshold we set. However, in choriocarcinoma, hypermethylation was strongly associated with gene downregulation (Figure 8).

When we looked at the numbers of up/downregulated and hypo/hypermethylated genes in some gene subsets, we found that DNA methylation does not have a differential effect on gene expression among imprinted genes compared to all genes. However, it has a substantial effect on villous trophoblast differentiation related (TBDE) [33] or predominantly placenta expressed genes (PPE) [16,26]. These latter effects are predominantly present in choriocarcinoma and less in complete moles (Figure 8).

The differentially methylated and expressed genes were divided into two main subsets based on whether their regulation by DNA methylation is likely (hypermethylated and downregulated or hypomethylated and upregulated) or not (hypermethylated and upregulated or hypomethylated and downregulated). Further analyses were restricted to the first set. This first set was further divided into six subsets based on the direction of expression/DNA methylation changes and whether the changes were observed both in complete mole and choriocarcinoma, or only in one of them. As we detected DNA methylation effects on gene expression among TBDE and PPE genes, we focused our further analyses on these genes in each subset. The genes in all subsets are listed in Supplementary Table S4, and their numbers in Table 1.

### 3.4. Genesis of Complete Moles and Choriocarcinoma Involve Shared and Distinct Pathways of Disease

**Figure 9:**
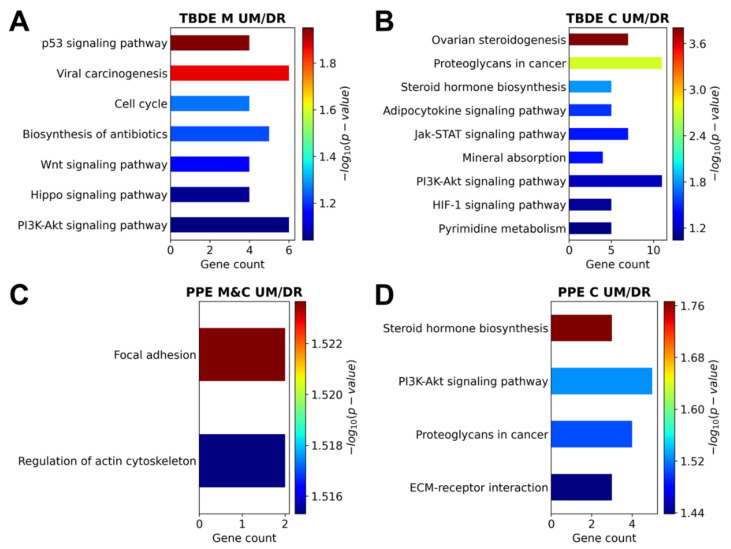
Analysis of KEGG pathways enriched in the subsets of trophoblast differentiation related or predominantly placenta expressed genes among differentially methylated and differentially expressed genes. (**A**) Pathways enriched in the set of 86 genes that are differentially expressed during trophoblast differentiation (TBDE) and are upmethylated and downregulated (UM/DR) in moles (M) but not in choriocarcinoma. (**B**) Pathways enriched in the set of 236 TBDE genes that are UM/DR in choriocarcinoma but not in moles. (**C**) Pathways enriched in the set of 7 placenta-specific (PPE) genes that are UM/DR both in moles and choriocarcinoma (M&C). (**D**) Pathways enriched in the set of 59 PPE genes UM/DR in choriocarcinoma.

As the last step, we examined the pathways enriched in the six subsets of genes in moles and/or in choriocarcinoma. Of importance, enriched pathways were mostly found among upmethylated and downregulated genes (see Supplementary Table S5). When the analyses were restricted to the TBDE (Supplementary Table S6) and PPE (Supplementary Table S7) genes, enriched pathways were only found in sets of upmethylated/downregulated in moles and in choriocarcinoma. Two pathways were shared between moles and choriocarcinoma among PPE genes in connection with focal adhesion and actin cytoskeleton; both are important in pathological processes in GTDs. In moles, seven pathways were enriched among TBDE genes, including cell cycle and differentiation regulatory pathways (p53-, Wnt-, Hippo-, and PI3K-Akt signaling), which are also essential in trophoblast differentiation and placental development. In choriocarcinoma, four pathways were enriched among PPE genes and nine pathways among TBDE genes, including those connected to steroid hormone biosynthesis, cancer proteoglycans, extracellular matrix–receptor interactions, hypoxic gene regulation, Jak-STAT, and PI3K-Akt signaling (Figure 9).

## 4. Discussion

### 4.1. Principal Findings of this Study

(1) DNA methylation increases with disease severity in gestational trophoblastic disease. (2) Differentially expressed genes are mainly upregulated in complete moles while predominantly downregulated in choriocarcinoma. (3) DNA methylation influences the gene expression of villous trophoblast differentiation-related or predominantly placenta-expressed genes. (4) The geneses of complete moles and choriocarcinoma involve shared and distinct pathways of disease.

### 4.2. Trophoblastic Gene Expression and Functions Are Severely Affected by Epigenetic Changes in GTDs

In a recent publication [18], we detected the enrichment of imprinted genes among differentially expressed genes as well as genes functioning in DNA methylation and the regulation of chromatin remodeling and gene expression in complete moles. Therefore, we hypothesized the dysregulation of placental gene regulation due to changes in DNA methylation and imprinting as the core of pathology in complete moles. Here, we extended this theory also to malignant gestational trophoblastic disease, choriocarcinoma, and examined this question with the use of high dimensional biology tools at the DNA, RNA, and protein levels, using our own and online accessible data.

We detected an increasing level of DNA methylation from normal (villous trophoblast, placenta) towards pathologic tissues with the escalation of clinical pathology, having the highest overall DNA methylation in invasive choriocarcinoma cells. In accordance, at the level of individual genes, moderate (Δβ, 0.25–0.5) DNA methylation was found in both GTDs while high (Δβ > 0.5) DNA methylation was almost exclusively found in choriocarcinoma. Of interest, most maternally imprinted sites were hypermethylated in both GTDs while most paternally imprinted sites only in choriocarcinoma, also reflecting to differences in the DNA methylation patterns in these GTDs. We found that CpG sites close to the TSS were more frequently and in a greater magnitude hypermethylated in choriocarcinoma, which suggests transcriptomic and functional consequences of this phenomenon.

Pointing to the potential causes of the observed DNA hypermethylation, we detected the dysregulation of members of the DNA methylase machinery in GTDs. In a complete mole transcriptome, the genome-wide de novo DNA methylase (*DNMT3A*) required also for parental imprinting [36] was downregulated, while *TET3* required for epigenetic reprogramming of the zygotic paternal DNA [37] was up-regulated along with four histone demethylases (*KDM4C*, lysine demethylase 4C; *KDM4D*; *KDM4E*; *KDM6B*) with developmental roles [38].

In choriocarcinoma cell transcriptome, the major de novo DNA methyltransferase active during early stage of embryonic development (*DNMT3B,* DNA methyltransferase 3 beta) [39,40] and its accessory protein (*DNMT3L,* DNA methyltransferase 3 like) required in complex with DNMT3A/B for embryonic de novo DNA methylation and genetic reprogramming [40,41] were upregulated. Interestingly, we found the non-significant upregulation of DNMT3B protein with immunohistochemistry in molar tissues, in accordance with gene expression data, which may suggest that choriocarcinoma immunostaining would be strongly positive for DNMT3B and encourages the investigation of its expression in choriocarcinoma tissue in a future study.

In addition, five histone lysine demethylases (*KDM2A*, lysine demethylase 2A, *KDM3A, KDM4B, KDM5A, KDM6A*) were downregulated. *KDM2A* has a key role in embryo development by regulating cell proliferation and survival [42]. *KDM3A* plays a pivotal role in regulating the expression of endoderm differentiation master genes [43], and in a regulatory circuit with hypoxia, HIF and MMP12, it is conserved and facilitates placental adaptations to environmental challenges [44]. *KDM4B* is required for maintaining stemness of trophoblastic stem cells by various protein interactors and epigenetic targets [45]. *KDM5A* is vital for normal zygotic genome activation and early embryo development [46]. *KDM6A* supports endoderm differentiation from embryonic stem cells and its overexpression improves the preimplantation development of the embryos [47].

The above-described changes in the expression of the DNA methylase machinery, including the non-significant upregulation of *DNMT3B* at the RNA level and moderately significant upregulation at the protein level in complete moles may be ‘sufficient’ only for limited pathological changes to the trophoblast compared to choriocarcinoma. In the latter condition, we observed the strong overexpression of de novo DNA methylases and marked downregulation of several histone lysine demethylases involved in early-stage embryo development and cell differentiation. These events are hypothesized to be responsible for the disturbances in chromatin remodeling after fertilization, including strong hypermethylation of the DNA, and the consequent lack of differentiation/dedifferentiation of the progenitors and associated transcriptomic changes involved in the carcinogenesis of trophoblastic cells. Indeed, this pattern resembles the DNA methylation patterns seen in TGCA data in which the more aggressive germ cell tumors were more highly DNA methylated, potentially emerging from less aggressive forms though a process that involved DNMT-mediated remethylation [48].

### 4.3. Shared and Distinct Trophoblastic/Placental Disease Pathways in GTDs

We found that most DE genes were upregulated in complete moles, irrespective of their methylation status, while most DE genes were downregulated in choriocarcinoma, in which downregulation was mostly associated with the hypermethylation of these genes. Of interest, when looking at subsets of hypermethylated and downregulated or hypomethylated and upregulated genes, DNA methylation turned out to have differential effects on gene expression among villous trophoblast differentiation (TBDE) related [33] or predominantly placenta expressed genes (PPE) [16,26] but not among imprinted genes, an effect which was present in both GTDs, especially in choriocarcinoma. The fact that most PPE genes were downregulated in both GTDs advocates that placenta-specific functions are turned down, although not to the same extent, in both diseases. Interestingly, since TBDE genes were marginally detected among DE genes in complete moles and these changed mostly in the same direction as during trophoblast differentiation (Figure 6), we could not find major villous trophoblast developmental defect for these functional changes in complete mole. In contrast, TBDE genes were strongly enriched among DE genes in choriocarcinoma cells, and their expression changed in the opposite direction as during trophoblast differentiation, suggesting that functional changes in choriocarcinoma are rooted in developmental problems of trophoblast differentiation. These differences in TBDE gene expression are also reflected by the difference in trophoblastic proteins’ maternal blood levels in moles and choriocarcinoma [49,50,51,52].

Due to their most functional relevance, we investigated biological pathways enriched in GTDs among the restricted set of upmethylated/downregulated TBDE or PPE genes. We found ‘focal adhesion’ and ‘regulation of actin cytoskeleton’ as common pathways of disease enriched in downregulated PPE genes in both GTDs. This is in line with changes in cytoskeletal elements and the expression of adhesion molecules critical for cell adhesion and migration during the invasive transformation of the trophoblast in GTDs [53,54,55,56].

In complete moles, seven biological pathways were impacted among TBDE genes, including ‘cell cycle’ and differentiation regulatory pathways essential for normal trophoblastic and placental development. The ‘Wnt signaling pathway’ is involved in the formation and function of extravillous trophoblasts including attachment and invasion, and failures in this signaling are associated with GTDs [57,58]. The ‘Hippo signaling pathway’ is involved in the maintenance of the human placental trophoblast epithelium by activating stemness factors and repressing genes that promote trophoblast cell fusion [59,60]. In conjunction with the ’cell cycle’, the ‘p53 signaling pathway’ induces cell cycle arrest, apoptosis, and senescence in the trophoblast [61,62]. The ‘PI3K-Akt signaling pathway’ plays an important role in extravillous trophoblast differentiation [63] and cell migration/invasion by modulating activities of matrix-degrading protease systems and ECM adhesion [63,64,65]. All these pathways are relevant for the known molecular pathology in complete moles, where trophoblasts have changed proliferative and invasive properties [66,67].

In choriocarcinoma cells, nine biological pathways were impacted among TBDE genes and four pathways among PPE genes. Three pathways were shared by both gene lists as potentially the most important ones, namely the ‘PI3K-Akt signaling pathway’, ‘steroid hormone biosynthesis’, and ‘proteoglycans in cancer’. Proteoglycans may interact with the ‘PI3K-Akt signaling pathway’ to affect trophoblast differentiation and cell migration/invasion [65,68]. Although the ‘PI3K-Akt signaling pathway’ pathway was also impacted in moles, several genes in this pathway were specifically affected only in choriocarcinoma. These include *EGFL6* (epidermal growth factor-like domain-containing protein-6), an EGF superfamily member related to tumor angiogenesis, growth, metastasis and progression that is also overexpressed in embryos [69]. A member of the epidermal growth factor (EGF) receptor family of receptor tyrosine kinases, *ERBB3* (ErbB2 receptor tyrosine kinase 3, HER3), which heterodimerizes with ErbB2/HER2 to function as an oncogenic unit and activate cell proliferation [70], was also upregulated. This is consistent with the increased expression of ErbB2 and ErbB3 during the malignant transformation of complete mole towards choriocarcinoma [71,72,73], and the general role of ErbB2/ErbB3 signaling in tumorigenesis by affecting autonomous cancer hallmarks (e.g., uncontrolled cell proliferation) and anti-tumor immune responses [70,74]. Of importance, several genes inhibited by *ERBB2/ERBB3* [75] were downregulated in choriocarcinoma, among which *ADAM19 (*Disintegrin And Metalloproteinase Domain-Containing Protein 19), *FN1* (fibronectin 1), and *FSTL3* (Follistatin Like 3) are involved in cell–cell and cell–matrix interactions and may impact cell adhesion during chorio-carcinogenesis. Interestingly, another growth factor (transforming growth factor-β1) pathway was strongly implicated in the pathogenesis and progression of GTDs [76,77], which suggests that disturbed growth factor signaling downstream of epigenetic changes may be in the center of disease in choriocarcinomas. In addition, ‘JAK-STAT signaling’, which is key for the cytokine- and growth factor-mediated modulation of cell proliferation and migration, was also found to be changed only in choriocarcinoma cells. This may be in line with the effects of a potentially immunosuppressive microenvironment compared to those in normal gestation on trophoblasts malignant behavior [78,79,80,81,82,83].

### 4.4. Strengths and Limitations of the Study

Our study utilized a set of high-dimensional biology tools to gain insights into the pathogenesis of GTDs at the levels of DNA methylation, gene expression, and biological pathway analyses. Additional strengths of our study included the use of strict clinical definitions and homogenous patient groups during clinical sample collections; internationally standardized histopathological examinations of molar pregnancies and placentas; expression profiling of candidate proteins on large tissue sets with tissue microarray and immunostaining followed by semi-quantitative immunoscoring and statistical analysis; and the use of leading bioinformatics tools for RNA-Seq, DNA methylation, and pathway analyses.

Limitations of our study included the non-availability of choriocarcinoma tissue DNA methylation or gene expression data which we complemented by using choriocarcinoma cell line data. In addition, we were not able to confirm choriocarcinoma gene expression at the protein level with our TMA immunostaining due to the bad quality of our choriocarcinoma tissue blocks. Due to the lack of other data, we had to combine various datasets (i.e., RNA-Seq, microarray), and could not directly compare molecular changes in complete moles and choriocarcinoma. Since we analyzed impacted biological pathways only among a restricted set of genes most relevant to trophoblastic disease, some altered pathways may have been missed.

## 5. Conclusions

Our data show that shared and disparate molecular pathways lead to the pathogenesis of complete moles and choriocarcinoma. More extensive expression changes in the DNA and histone methylation machinery primes robust epigenetic and downstream signaling pathway alterations in choriocarcinoma, leading to the malignant transformation of the trophoblast. Our findings provide new insights into these disease pathways in GTDs and may help in designing new diagnostic and therapeutic tools.

## Figures and Tables

**Table 1:** Numbers of genes in various subsets of differentially DNA methylated and expressed genes. Subsets of genes were defined based on the direction of DNA methylation and expression changes (UM: upmethylated, DM: downmethylated, UR: upregulated, DR: downregulated) in moles (M), choriocarcinoma (C), and their intersections (M&C). The fractions of genes in each group are visualized in Figure 7, where the same abbreviations are used.

Subset	Number of Genes	Imprinted (204)	PPE (164)	TBDE (1937)
M&C DM/UR	1	0	0	0
M&C UM/DR	34	0	7	20
M DM/UR	405	4	1	31
C DM/UR	17	0	0	9
M UM/DR	307	4	10	86
C UM/DR	714	12	59	236
M&C DM/DR	0	0	0	0
M&C UM/UR	9	1	0	4
M DM/DR	111	3	4	22
M UM/UR	723	9	6	73
C DM/DR	25	0	0	12
C UM/UR	298	4	0	91
Total	2644	37	87	584

## Data Availability

USC RNA-Seq data was deposited to the Gene Expression Omnibus (GEO) database according to the MIAME guidelines (accession number: GSE138250). All other data was downloaded from publicly archived datasets as detailed in Section 2.4.

## Supplementary Materials

The following are available online at https://www.mdpi.com/article/10.3390/biomedicines9121935/s1, Table S1: Public Data Table, Table S2: Differential expressed genes between choriocarcinoma and villous trophoblast, Table S3: Identified DM genes in placenta, moles and choriocarcinoma, Table S4: Differential expressed and methylated genes in complete mole—placenta and choriocarcinoma—villous trophoblast comparisons, Table S5: Pathways enriched in differentially methylated and expressed genes, Table S6: Pathways enriched in differentially methylated and expressed TBDE genes, Table S7: Pathways enriched in differentially methylated and expressed PPE genes.

## Appendix A

**Table A1:** Demographic characteristics of patients in the immunostaining study.

	GTD Tissue Samples (*n* = 17)	Placenta Tissue Samples (*n* = 24)
Race/ethnicity ^a^		
Hispanic	18 (78%)	
Non-Hispanic white	2 (9%)	24 (100%)
Asian	0	
African-American	0	
Unknown/other	3 (13%)	
Age (years) at procedure ^b^	31.9 ± 9.5	28.8 ± 6.2
Comorbidities		
Hypertension ^a^	1 (4%)	
Diabetes ^a^	1 (4%)	
β-hCG (mIU/mL) at procedure ^c^	185,277 (70,349–387,812)	
Histologic diagnosis ^a^		
Complete hydatidiform mole (CHM)	17 (100%)	0 (0%)
p57 immunostaining confirmation of CHM	17 (100%)	
Gestational age at the procedure (days) ^b^	61.65 ± 16.97	62.79 ± 12.30

A total of 17 samples altogether; one case with two specimens at the same time; another case with three specimens at different time points. ^a^ Number (percentage). ^b^ Mean ± SD. ^c^ Median (IQR). Cyclin-dependent kinase inhibitor 1C, p57; human chorionic gonadotropin, hCG; gestational trophoblastic disease, GTD; tissue microarray, TMA.

**Table A2:** Immunostaining conditions.

Primary Antibody (Concentration/Dilution, Distributor)	Detection Antibody (Distributor)	Detection System (Distributor)
mouse monoclonal anti-human p57 antibody (1:3000) (code: MA5-11309, Thermo Fisher Scientific, Waltham, MA, USA)	Novolink detections system (Leica-Novocastra, Wetzlar, Germany)	Novolink DAB/substrate kit (Leica-Novocastra, Wetzlar, Germany)
rabbit polyclonal anti-human DNMT3B antibody (1:100) (code: LS-C352124, LSBio, Seattle, WA, USA)		
